# Combinatorial refinement of thin-film microstructure, properties and process conditions: iterative nanoscale search for self-assembled TiAlN nanolamellae

**DOI:** 10.1107/S1600576716017258

**Published:** 2016-12-01

**Authors:** J. Zalesak, J. Todt, R. Pitonak, A. Köpf, R. Weißenbacher, B. Sartory, M. Burghammer, R. Daniel, J. Keckes

**Affiliations:** a Department Metallkunde und Werkstoffprüfung, Montanuniversität Leoben, 8700 Leoben, Austria; b Department Materialphysik, Montanuniversität Leoben, 8700 Leoben, Austria; c Böhlerit GmbH and Co KG, Kapfenberg, 8605, Austria; d Materials Center Leoben GmbH, 8700 Leoben, Austria; e ESRF, 38043 Grenoble, France

**Keywords:** X-ray nanodiffraction, thin films, nanomaterials, combinatorial search

## Abstract

Because of the tremendous variability of crystallite sizes and shapes in nano­materials, it is challenging to assess the corresponding size–property relationships and to identify microstructures with particular physical properties or even optimized functions. This task is especially difficult for nanomaterials formed by self-organization, where the spontaneous evolution of microstructure and properties is coupled. In this work, two compositionally graded TiAlN films were (i) grown using chemical vapour deposition by applying a varying ratio of reacting gases and (ii) subsequently analysed using cross-sectional synchrotron X-ray nanodiffraction, electron microscopy and nanoindentation in order to evaluate the microstructure and hardness depth gradients. The results indicate the formation of self-organized hexagonal–cubic and cubic–cubic nanolamellae with varying compositions and thicknesses in the range of ∼3–15 nm across the film thicknesses, depending on the actual composition of the reactive gas mixtures. On the basis of the occurrence of the nanolamellae and their correlation with the local film hardness, progressively narrower ranges of the composition and hardness were refined in three steps. The third film was produced using an AlCl_3_/TiCl_4_ precursor ratio of ∼1.9, resulting in the formation of an optimized lamellar microstructure with ∼1.3 nm thick cubic Ti(Al)N and ∼12 nm thick cubic Al(Ti)N nanolamellae which exhibits a maximal hardness of ∼36 GPa and an indentation modulus of ∼522 GPa. The presented approach of an iterative nanoscale search based on the application of cross-sectional synchrotron X-ray nanodiffraction and cross-sectional nanoindentation allows one to refine the relationship between (i) varying deposition conditions, (ii) gradients of microstructure and (iii) gradients of mechanical properties in nanostructured materials prepared as thin films. This is done in a combinatorial way in order to screen a wide range of deposition conditions, while identifying those that result in the formation of a particular microstructure with optimized functional attributes.

## Abstract 2

A novel iterative combinatorial nanoscale search based on the application of cross-sectional synchrotron X-ray nanodiffraction and cross-sectional nanoindentation is used to refine the relationship between deposition conditions, microstructure and properties of nanostructured TiAlN thin films. Using three iterative steps, a nanolamellar TiAlN thin film with a maximal hardness of ∼36 GPa is developed.

## 1. Introduction   

In nanomaterials with crystallite sizes below 100 nm, the physical properties significantly differ from those of bulk materials. The properties of nanomaterials can be correlated with (i) the variation of lattice spacing and corresponding chemical bonding nature, especially at nanocrystal surfaces, and/or (ii) the large volume fraction of grain boundaries in polycrystalline materials (Arzt, 1998 ▸; Gleiter, 1989 ▸). Owing to the tremendous variability of crystallite sizes and shapes in nanomaterials, it is not trivial to quantitatively evaluate the correlation between crystallite sizes and nanomaterial properties, the ‘size effect’ of physical properties.

Hard nanocrystalline thin films based, for example, on TiN, CrN and TiAlN (used in metal cutting applications) represent a typical example of a technological system in which small crystallites decisively contribute to enhanced functional properties such as high hardness, high wear resistance and toughness (Veprek & Argon, 2002 ▸; Zhang *et al.*, 2003 ▸). In the case of thin films synthesized using physical and chemical vapour deposition (PVD and CVD), not only (i) specific phases but also (ii) the particular microstructure and (iii) the residual stress state play decisive roles in the functional behaviour of the film (Veprek *et al.*, 2003 ▸; Zhang *et al.*, 2007 ▸). Therefore the microstructural design is important, especially in the case of hard films where fracture toughness can be significantly enhanced by grain boundary engineering (Mayrhofer *et al.*, 2006 ▸; Zhang *et al.*, 2007 ▸).

In the case of PVD thin films prepared by magnetron sputtering, it is possible to control the microstructural development to a certain extent by varying the deposition parameters and conditions such as temperature, deposition rate, substrate bias and direction of incoming particles (Mayrhofer *et al.*, 2006 ▸). In particular, the application of intense ion bombardment can be used to synthesize materials of very complex microstructure far from thermodynamic equilibrium. However, in thin films whose microstructure is formed as a result of self-organization phenomena at or near thermodynamic equilibrium, like in the case of CVD thin films, it is not trivial (i) to identify deposition conditions resulting in the desired microstructure and subsequently (ii) to effectively tune functional properties (Choy, 2003 ▸).

In the context of characterizing very local structural properties of nanocrystalline thin films, the recently introduced technique of cross-sectional X-ray nanodiffraction has demonstrated the ability to assess volume-averaged cross-sectional distributions of phases, microstructure and stresses in thin films (Keckes *et al.*, 2012 ▸; Stefenelli *et al.*, 2013 ▸). This new approach opened the way to correlate X-ray diffraction (XRD) data with the results from other techniques like cross-sectional nanoindentation, to perform correlative nanoanalytics in order to determine structure–property relationships at the nanoscale. By achieving this milestone, it was for the first time possible to evaluate the role of distinct microstructural features in the mechanical response of thin films, as demonstrated in our previous study on a graded TiAlN thin film prepared by PVD (Zalesak *et al.*, 2016 ▸).

The present work (i) introduces a new iterative combinatorial nanoscale approach which was used to (ii) characterize microstructured Ti*_x_*Al_1−*x*_N thin films prepared by CVD as a result of a self-organized growth from a gas phase. The specific film microstructure already introduced in our previous studies possesses a high volume fraction of self-assembled nanolamellae, which are formed as a result of oscillating chemical reactions during the growth (Keckes *et al.*, 2013 ▸; Todt *et al.*, 2014 ▸). The aim of this work is to correlate the thickness and the composition of the nanolamellae, the local mechanical properties, and the deposition conditions in order to identify process conditions resulting in the formation of a nano­lamellar film with a maximal hardness. For this purpose the combinatorial approach based on the combination of cross-sectional synchrotron X-ray nanodiffraction and cross-sec­tional nanoindentation was developed and used to characterize compositionally graded Ti*_x_*Al_1−*x*_N films with varying nanolamellar periods.

## 2. Experimental   

### 2.1. Thin-film deposition   

The Ti*_x_*Al_1−*x*_N thin films characterized in this work were prepared in a Bernex MT-CVD-300 medium-temperature reactor using the process gases AlCl_3_, TiCl_4_, NH_3_ and N_2_ with H_2_ as carrier gas (Todt *et al.*, 2014 ▸). For the deposition of the films on WC–Co (6 wt%) cemented carbide substrates, a deposition temperature of 1073 K and a total process pressure of 2.5 kPa were applied. For the deposition of graded films denoted as A and B, varying ratios of precursors AlCl_3_ and TiCl_4_ in the range of 0.6–2.75 were used, as shown in Table 1 ▸. In order to evaluate the influence of (i) the substrate and (ii) the sequence of the applied AlCl_3_/TiCl_4_ ratios on the formation of the self-assembled thin films, films A and B were synthesized with decreasing and increasing AlCl_3_/TiCl_4_ ratios (Table 1 ▸), respectively. For the deposition of the film denoted as C, a constant AlCl_3_/TiCl_4_ ratio of 1.9 was selected.

### 2.2. Cross-sectional X-ray nanodiffraction   

From all three thin films A, B and C, cross-sectional slices consisting of the substrate and the film with a slice thickness of ∼40 µm, a length of ∼4 mm and a height of ∼2 mm were prepared by mechanical polishing. The slices were analysed in transmission wide-angle diffraction geometry at the nanofocus extension of the ID13 beamline at the European Synchrotron Radiation Facility (ESRF) in Grenoble, France (Riekel *et al.*, 2010 ▸). A monochromatic X-ray beam of energy 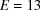
 keV was focused using a Fresnel zone plate, providing a beam with horizontal and vertical full widths at half-maximum (FWHMs) of ∼100 and ∼96 nm, respectively. The sample–detector distance was ∼9 cm. During the measurement, the thin-film slices were moved in the beam, applying a scanning step size (along the vertical *z* axis) of 100 nm, and Debye–Scherrer rings were collected for all cross-sectional thin-film *z* positions. The two-dimensional powder diffraction data were then treated using the software *FIT2D* (Hammersley, 2016 ▸) in order to obtain intensity *I versus* Bragg angle θ plots 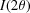. 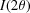 data obtained for various thin-film depths *z* were used to compile three-dimensional plots 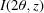. Other details describing cross-sectional X-ray nanodiffraction can be found in our previous reports (Keckes *et al.*, 2012 ▸; Bartosik *et al.*, 2013 ▸).

### 2.3. Cross-sectional nanoindentation   

Cross-sectional characterization of the mechanical properties of mechanically polished Ti*_x_*Al_1−*x*_N thin-film cross sections was performed using an atomic force microscope (Veeco Dimension 3100), which was equipped with a sharp diamond cube-corner tip controlled by a Hysitron Triboscope transducer in load–displacement mode. For the indenter calibration, monocrystalline sapphire (0001) was used. For all indents, a maximal load of 400 µN was applied (Zalesak *et al.*, 2016 ▸). The films were indented at various cross-sectional positions, and the measured load–displacement curves were used to evaluate the hardness 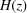 and indentation modulus 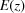 at various film depths *z* according to the Oliver & Pharr (1992 ▸) method.

### 2.4. Cross-sectional electron microscopy   

The cross-sectional morphologies of the films were investigated using a Zeiss AURIGA scanning electron microscope equipped with an energy-dispersive X-ray spectroscopy (EDX) unit in order to evaluate concentration profiles (using built-in sensitivity factors) for Ti and Al across the films’ thicknesses.

In order to obtain complementary information on the microstructural properties of the films, transmission electron microscopy (TEM) was employed. For this purpose, cross-sectional TEM lamellae of samples A and C were machined using a focused ion beam milling workstation (Orsay Physics Cobra Z-05) attached to a Zeiss Auriga 60 Crossbeam field emission gun scanning electron microscope. The cross-sectional TEM lamellae were glued onto a Cu TEM holder and subsequently polished to a thickness of about 30 nm. The TEM characterization was performed using an image-side-corrected JEOL JEM-2100F TEM system operated at 200 keV in both scanning transmission electron microscopy (STEM) and TEM mode. For the STEM imaging, a 0.7 nm spot size and a high-angle annular dark-field detector were used in order to visualize the *Z* contrast. The main aim of the TEM analysis was to resolve the nanoscale morphology and microstructure of the self-organized nanolamellae formed in the films.

## 3. Results   

### 3.1. Thin film A   

**Figure 1:**
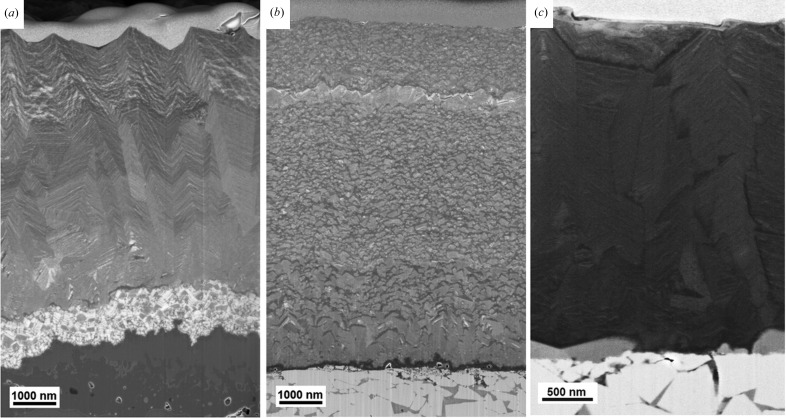
SEM micrographs showing cross sections of the analysed thin films A (*a*), B (*b*) and C (*c*) on WC–Co substrates. In films A and B, the variation of the deposition conditions during the film growth resulted in the formation of graded microstructure.

As already indicated in §2.1, film A was prepared by using varying deposition conditions, namely the precursor ratio AlCl_3_/TiCl_4_ was changed in steps of about 0.1 during the deposition process (*cf.* Table 1 ▸). In the region near the film–substrate interface, with a thickness of about 1.5 µm, film A possessed the composite grain microstructure (with small dark grains of various shapes and sizes embedded in a light matrix) visible in the scanning electron microscopy (SEM) image in Fig. 1 ▸(*a*). At distances greater than 1.5 µm from the interface, a columnar grain microstructure with a chevron-like morphology developed. SEM-EDX analysis indicated that the Ti/Al atomic concentration ratio in film A increased linearly towards the surface, in accordance with the variation of gas flow ratio, as presented in Table 1 ▸.

**Figure 2:**
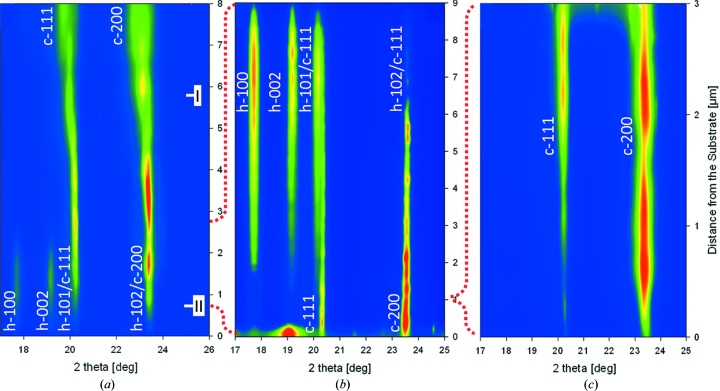
Phase plots 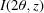 obtained using cross-sectional X-ray nanodiffraction from samples A (*a*), B (*b*) and C (*c*). Labels indicate the presence of hexagonal (h) and cubic (c) phases with the corresponding diffraction peaks. The transitions from hexagonal to cubic and from cubic to hexagonal phases occur at distances of ∼2.3 and 1 µm from the interface in (*a*) and (*b*), respectively. In the monolithic film C, only cubic peaks were observed. Positions I and II in (*a*) indicate regions for which the cross-sectional TEM micrographs in Figs. 3(*a*) and 3(*b*) and Figs. 4(*a*) and 4(*b*) were recorded, respectively. The dotted lines schematically indicate the iteratively narrowing phase, microstructure and process windows.

The X-ray nanodiffraction phase plot 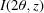 in Fig. 2 ▸(*a*) indicates cross-sectional changes in the phase occurrence and in the diffraction peak morphology in film A. At a distance of ∼0–2.3 µm from the film–substrate interface, diffraction peaks of hexagonal (h) and cubic (c) phases, h-­100, h-002, h-101, h-102 and c-111, c-200, can be identified. At a distance of 2.3–8 µm from the interface, the hexagonal peaks diminish and only cubic phase peaks were recorded (*cf.* Fig. 2 ▸ *a*). Additionally, the FWHMs of the cubic peaks and the lattice parameter of the cubic phase significantly increased towards the surface. For instance, the FWHMs of the c-200 reflections and the evaluated lattice parameters changed from 0.179 to 1.19° and from 0.4177 to 0.424 nm between the distances of 2.3 and 8 µm from the interface, respectively.

**Figure 3:**
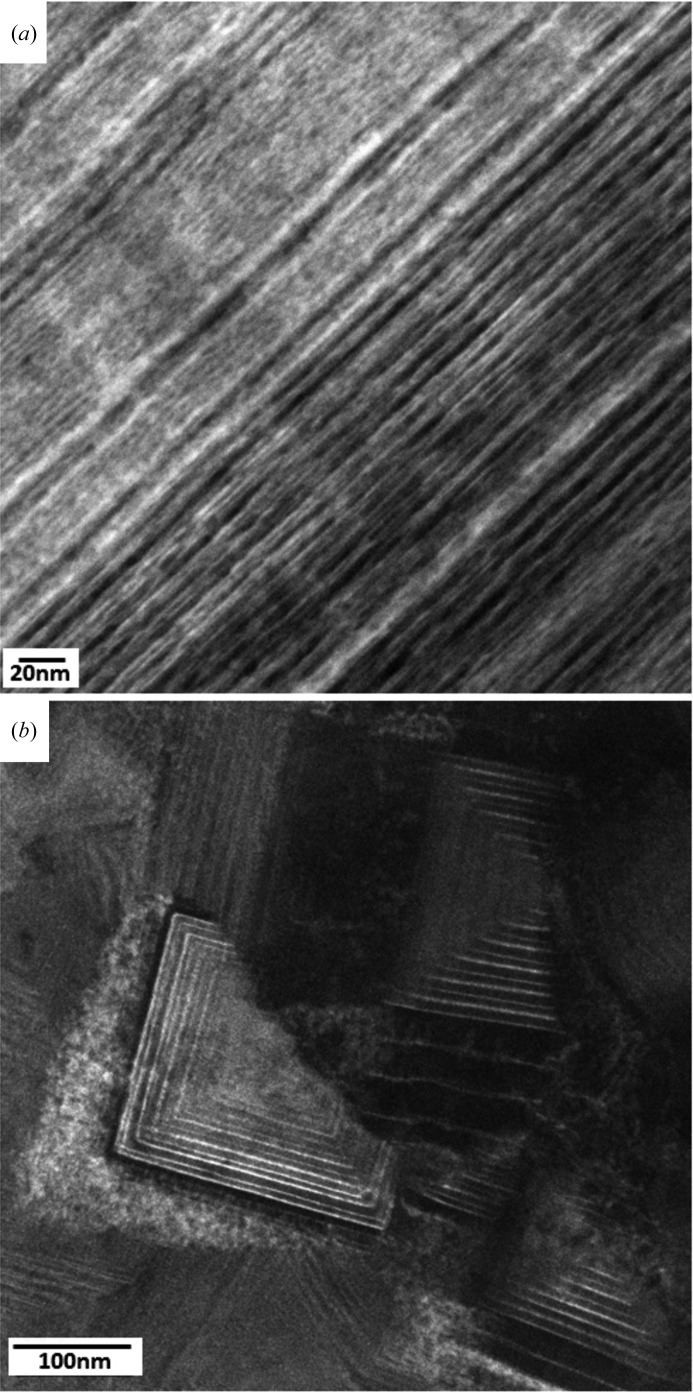
Bright-field TEM micrographs (*a*) and (*b*) were collected from film A at the cross-sectional positions denoted as I and II in Fig. 2(*a*). The bright and dark lines represent Al(Ti)N and Ti(Al)N nanolamellae, respectively, whose thicknesses change across the film cross section in the range of ∼3–15 nm.

The cross-sectional positions denoted as I and II in Fig. 2 ▸(*a*) indicate the approximate positions of the thin-film regions for which the cross-sectional TEM micrographs in Figs. 3 ▸(*a*) and 3 ▸(*b*), respectively, were recorded. The bright-field TEM images in Fig. 3 ▸, as well as additional TEM studies, documented that across the whole film thickness the film possesses a nanolamellar internal grain microstructure with a lamellar thickness of ∼3–15 nm, visible as bright and dark lines in Fig. 3 ▸.

**Figure 4:**
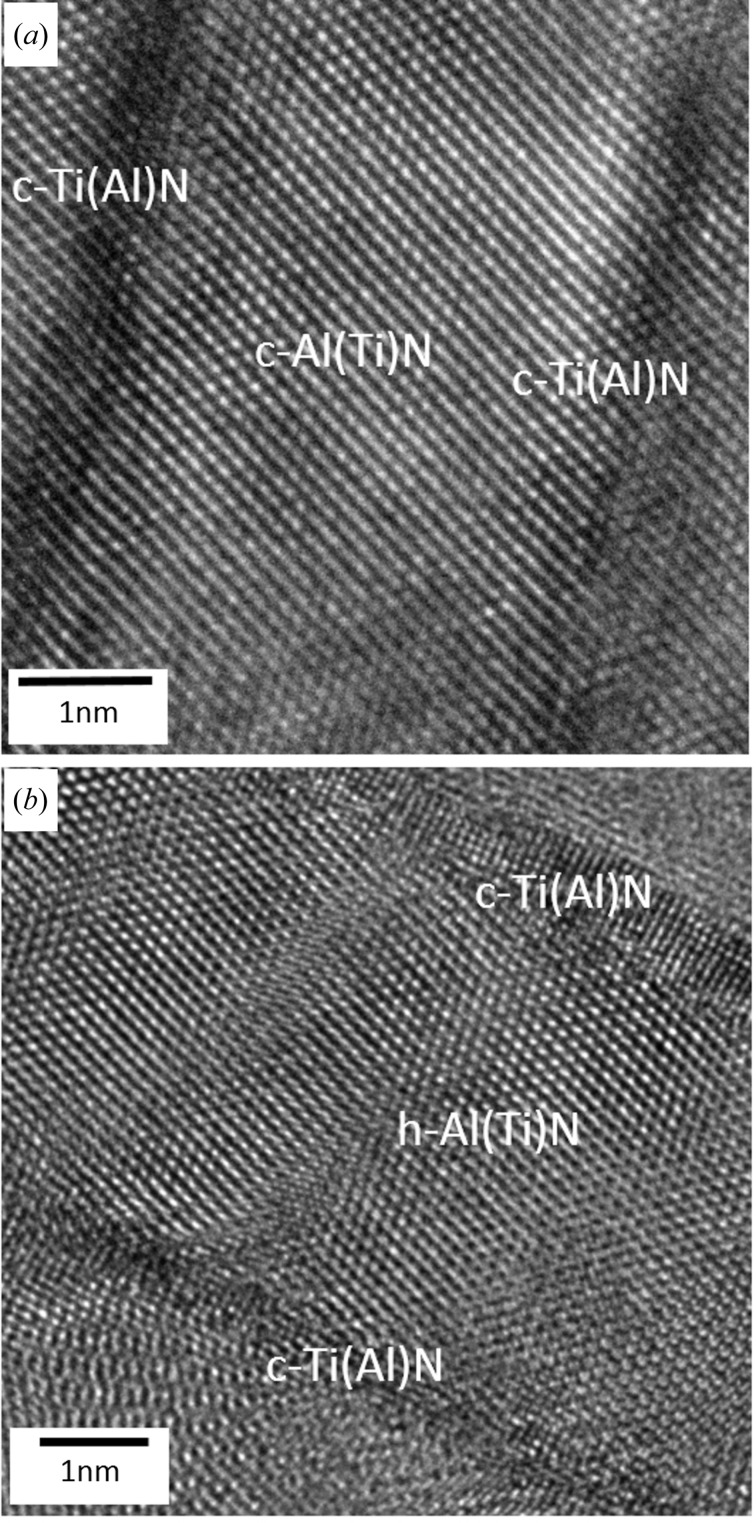
HR-TEM micrographs showing alternating (*a*) coherent c-Al(Ti)N and c-Ti(Al)N nanolamellae and (*b*) incoherent h-Al(Ti)N and c-Ti(Al)N nanolamellae in film A at positions I and II (*cf.* Fig. 2*a*).

The high-resolution (HR) TEM micrographs in Figs. 4 ▸(*a*) and 4 ▸(*b*) were also collected, respectively, from the approximate cross-sectional positions denoted as I and II in Fig. 2 ▸(*a*). The HR-TEM data as well as EDX analyses (not presented here) showed that the alternating bright and dark lamellae from Fig. 3 ▸ actually consisted of AlN and TiN phases with a small fraction of Ti and Al atoms, respectively, at the substitutional positions, and therefore these will be further denoted as Al(Ti)N and Ti(Al)N lamellae. In all three films, the overall nitrogen concentration was approximately stoichiometric. Electron energy loss spectroscopy (not presented here), however, showed an oscillation of nitrogen concentration across the lamellae.

The HR-TEM analysis of the film (Fig. 4 ▸ *b*) from position II (*cf.* Fig. 2 ▸ *a*) indicated (i) that in this region film A consisted of cubic and hexagonal nanolamellae, giving rise to the hexagonal and cubic peaks in Fig. 2 ▸(*a*), and (ii) that the interfaces between hexagonal Al(Ti)N and cubic Ti(Al)N lamellae were incoherent. HR-TEM data collected from film position I (*cf.* Fig. 2 ▸ *a*) indicated that hexagonal lamellae were no longer present in the film, which is in agreement with the XRD data from Fig. 2 ▸(*a*). This region of film A consisted of predominantly coherent c-Al(Ti)N and c-Ti(Al)N lamellae, shown in detail in Fig. 4 ▸(*a*). The phase plot in Fig. 2 ▸(*a*) and the TEM micrographs in Fig. 4 ▸ suggest that, as a result of the increase in the relative amount of TiCl_4_ precursor in the deposition chamber, only alternating c-Al(Ti)N and c-Ti(Al)N lamellae were formed in the film at distances greater than 2.3 µm from the interface.

**Figure 5:**
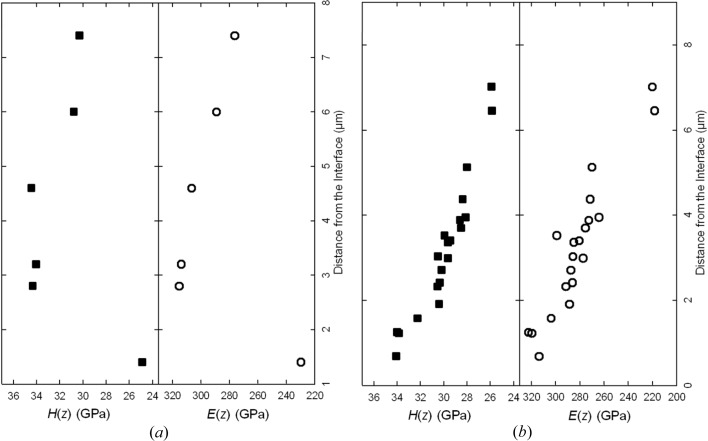
Cross-sectional dependencies of indentation hardness 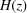 and indentation modulus 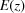 in samples A (*a*) and B (*b*), possessing maxima at 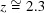
 µm and 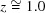
 µm, respectively.

In order to correlate the microstructural information from Figs. 1 ▸–4 ▸ ▸ ▸ with the local mechanical properties, cross-sectional nanoindentation experiments were performed on film A. In Fig. 5 ▸(*a*), the depth dependencies of the indentation hardness 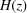 and indentation modulus 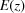 are presented. The results show that the maximum hardness of ∼35 GPa, as well as the maximum indentation modulus of ∼522 GPa, corresponds to the region of film A located at a distance ranging from ∼2.3 to ∼4.5 µm from the substrate interface, where the hexagonal Al(Ti)N phase continuously changed to the cubic Al(Ti)N phase (*cf*. Fig. 2 ▸ *a*). Further film growth, dominated by the cubic Al(Ti)N phase, resulted in a slight decrease in hardness and indentation modulus. This observation obviously indicates that the cubic phase is beneficial for the mechanical properties of the film, as already reported by others (Mayrhofer *et al.*, 2003 ▸; PalDey & Deevi, 2003 ▸). The formation of the film microstructure with the maximum hardness is therefore related to the extinction of the h-Al(Ti)N phase, while the Al content of the layer remains comparatively high at a distance of 2.3–4.5 µm from the interface (*cf.* Figs. 2 ▸ *a* and 5 ▸ *a*).

Since the main aims of this study were (i) to clarify the microstructure–property relationship and (ii) to identify deposition parameters resulting in the highest hardness, a new graded sample denoted as B was produced using a much narrower window of AlCl_3_/TiCl_4_ precursor ratio (*cf.* Table 1 ▸). Thus a higher resolution for the precise identification of optimal microstructure, hardness and, ultimately, deposition process parameters was obtained.

### 3.2. Thin film B   

As documented in Table 1 ▸, the precursor ratio in film B was increased during the deposition (contrary to film A) and therefore the variation of microstructure, phases and mechanical properties is expected to be inverted.

The cross-sectional phase plot 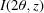 from sample B presented in Fig. 2 ▸(*b*) indicates that, at a distance of ∼0–1 µm from the substrate, only cubic diffraction peaks [originating from c-Al(Ti)N and c-Ti(Al)N lamellae] were detected. The formation of h-Al(Ti)N lamellae was observed at a distance of ∼1 µm and farther from the film–substrate interface. The corresponding depth dependencies of the indentation hardness 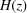 and elastic modulus 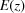 of the cross-sectional nanoindentation experiment performed on film B are shown in Fig. 5 ▸(*b*). The maximum hardness and indentation modulus were observed at a distance of ∼1 µm from the interface, which corresponds to the cubic–hexagonal Al(Ti)N transition shown in the XRD data in Fig. 2 ▸(*b*). In the film region dominated by the hexagonal phases, both hardness and elastic modulus decrease with increasing volume fraction of the hexagonal lamellae. SEM-EDX analysis indicated that the Ti/Al atomic concentration ratio in film B decreased linearly towards the surface, in accordance with the variation of gas flow ratio, as presented in Table 1 ▸.

A comparison of (i) XRD data 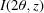 from Fig. 2 ▸(*b*), (ii) the cross-sectional distributions of hardness 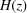 and indentation modulus 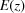 from Fig. 5 ▸(*b*), and (iii) the recorded deposition process parameters allowed us to identify an AlCl_3_/TiCl_4_ precursor ratio of ∼1.9 as the most favourable process gas composition for the formation of (i) cubic Al(Ti)N nanolamellae with a maximum thickness of ∼12 nm and (ii) subsequently an AlTiN thin film with maximum hardness and elastic modulus. Finally, after the second iteration step, it was possible to synthesize a monolithic thin film with optimized mechanical properties (§3.3).

### 3.3. Thin film C   

Sample C was synthesized using a constant AlCl_3_/TiCl_4_ precursor ratio of ∼1.9, identified from the analysis of the graded sample B (§3.2). For this reason, the sample possessed constant composition across the whole thickness, as documented by the cross-sectional phase plot 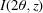 in Fig. 2 ▸(*c*). The intensity variation of the diffraction peaks in Fig. 2 ▸(*c*) was caused by the crystallographic texture gradients associated with specific film evolution during the growth. The observed c-111 and c-200 peaks in Fig. 2 ▸(*c*) indicate a purely cubic thin-film nature.

**Figure 6:**
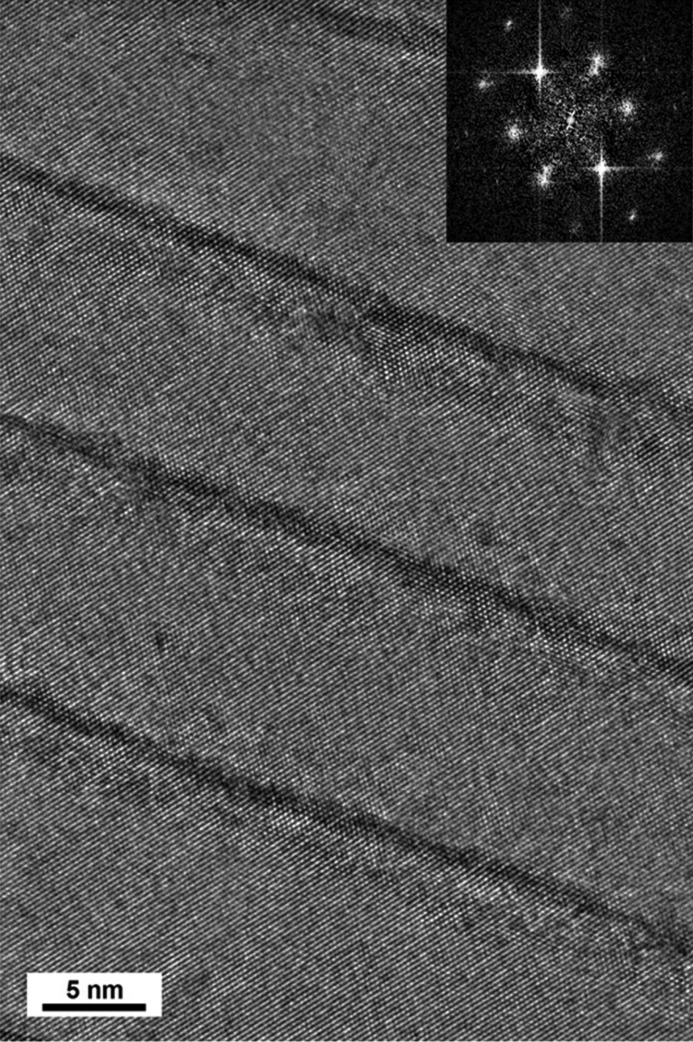
An HR-TEM micrograph showing alternating coherent c-Al(Ti)N and c-Ti(Al)N nanolamellae in film C. An inset with a fast Fourier transformation indicates the presence of only the cubic phase within the probed volume.

SEM-EDX analysis indicated that the atomic concentration ratio of Ti/Al was 20:80 (Todt *et al.*, 2016 ▸). The HR-TEM micrograph of film C in Fig. 6 ▸ documents that the film consisted of alternating cubic Al(Ti)N and cubic Ti(Al)N nanolamellae with thicknesses of ∼12 and ∼1.25 nm, respectively, in which interfaces between the lamellae were coherent, similar to the interfaces of the cubic region II of film A (Fig. 4 ▸ *b*). Hardness and indentation modulus characterization of the film surface provided values of 36.6 and 522 GPa, respectively. It might be expected that film C would exhibit a certain cross-sectional variation of mechanical properties and internal grain microstructure, which can be attributed to the evolution of the CVD growth kinetics during the otherwise constant process conditions. The evaluation of this effect is, however, not in the focus of this combinatorial work.

## 4. Discussion   

By the application of cross-sectional X-ray nanodiffraction, cross-sectional nanoindentation and TEM, it was possible to iteratively refine deposition conditions, resulting in the formation of an optimized TiAlN film with a specific nanolamellar microstructure composed of coherently arranged c-Ti(Al)N and c-Al(Ti)N nanolamellae with thicknesses of ∼1.25 and ∼12 nm, respectively. In particular, the presence of the c-Al(Ti)N phase was identified as very beneficial for the film’s mechanical properties.

The phase plots in Figs. 2 ▸(*a*) and 2 ▸(*b*) document that an increase in the relative amount of TiCl_4_ precursor in the deposition chamber gives rise to the formation of alternating cubic Al(Ti)N and Ti(Al)N lamellae in the films. Note that the AlN phase can exist under ambient conditions only in the hexagonal modification since the cubic polytype is metastable (Mayrhofer *et al.*, 2003 ▸). A first-order phase transition from hexagonal to cubic structure is observed for AlN at high pressure (Ueno *et al.*, 1992 ▸). It is thus evident from the XRD diffraction data (Fig. 2 ▸ *a*) and from TEM investigations (Fig. 4 ▸ *a*) that cubic Ti(Al)N sublayers stabilized the Al(Ti)N lamellae and promoted the growth of a cubic (very probably metastable) Al(Ti)N phase in the films. This effect can be considered as epitaxial stabilization. The effect has already been reported in superlattice structures of CrN–AlN (Lin *et al.*, 2009 ▸; Schlögl *et al.*, 2013 ▸), TiN–AlN (Setoyama *et al.*, 1996 ▸; Madan *et al.*, 1997 ▸) and other (Lattemann *et al.*, 2002 ▸; Söderberg *et al.*, 2006 ▸; Stueber *et al.*, 2009 ▸) thin films deposited using molecular beam epitaxy and magnetron sputtering. In those cases, cubic AlN sublayers with a thickness in the nanometre rage were strain stabilized by surrounding cubic CrN or TiN sublayers. In the present case, however, the formation of cubic Al(Ti)N sub-lamellae occurs spontaneously, probably as a result of oscillating chemical reactions at the surface of the thin film during its growth (Bartsch *et al.*, 1992 ▸). The formation of the cubic Al(Ti)N phase is therefore a result of the specific deposition process giving rise to the formation of the distinct nanolamellar microstructure. This argument is supported also by the fact that the interfaces between the c-Al(Ti)N and c-Ti(Al)N nanolamellae are coherent (*cf.* Fig. 4 ▸ *a*). The presence of ternary c-Al(Ti)N and c-Ti(Al)N phases can also serve as an explanation for the formation of relatively thick cubic Al(Ti)N nanolamellae. This is because the relatively small concentration of Ti or Al atoms alloyed into AlN and TiN, respectively, will increase and reduce the Al(Ti)N and Ti(Al)N lattice parameters, respectively, which subsequently reduces the lattice strain needed for the stabilization of the cubic Al(Ti)N phase. Thus the cubic Al(Ti)N lamellae may have significantly greater thickness than pure c-AlN layers in CrN/AlN mutlilayers (Madan *et al.*, 1997 ▸; Setoyama *et al.*, 1996 ▸; Schlögl *et al.*, 2013 ▸).

The crystallographic orientation relationship at the interfaces between incoherent cubic–hexagonal and coherent cubic–cubic lamellae from Fig. 4 ▸ can be expressed as c-Ti(Al)N(110)||h-Al(Ti)N(10.0) and c-Ti(Al)N(100)||c-Al(Ti)N(100), respectively. The reported literature values of c-AlN, h-AlN and c-TiN lattice parameters are ∼0.406, ∼0.437 and ∼0.424 nm, respectively (Kohn & Sham, 1965 ▸; Christensen & Gorczyca, 1994 ▸). Consequently, an epitaxial growth of TiN on h-AlN and c-AlN (and *vica versa*, as observed in Fig. 4 ▸) results in a lattice mismatch of ∼4.2 and ∼3.1%. Such high mismatches lead usually to high interfacial energy and the formation of misfit dislocations. Therefore, in the case of incoherent c-Ti(Al)N/h-Al(Ti)N interfaces (*cf.* Fig. 4 ▸ *b*), a relatively large density of dislocations was observed (*cf.* Fig. 4 ▸ *b*), contrary to a very small number of defects at the coherent c-Ti(Al)N/h-Al(Ti)N interfaces (*cf.* Fig. 4 ▸ *a*). The latter can be interpreted as the result of a smaller lattice mismatch between c-TiN and c-AlN and the presence Al and Ti atoms in the substitutional positions in the respective phases.

The correlation of the highest hardness with the greatest thickness of the cubic Al(Ti)N lamellae of 12 nm can be explained by the volume increase of ∼26% during the cubic to hexagonal phase transformation (Schlögl *et al.*, 2013 ▸). It can be expected that during an indentation experiment, the cracks propagating in the brittle material under the indenter tip modify the local strain state and generate new free surfaces at the crack tip, allowing for a localized cubic to hexagonal phase transformation. Subsequently, the volume increase may result (i) in the formation of compressive strains at the crack tip, as well as (ii) in the absorption of the crack energy and (iii) in the crack deceleration, deflection or even termination. Whenever a free volume is generated at the crack tip, accompanied by a phase transformation, the effect of the volume increase should be larger in the vicinity of relatively thick cubic Al(Ti)N nanolamellae than in very thin cubic Al(Ti)N lamellae neighbouring comparatively thick cubic Ti(Al)N nanolamellae.

Another, maybe simpler, explanation might be that the nanolamellar composite can benefit most from the improved mechanical properties of cubic Al(Ti)N over its hexagonal counterpart, when the volume fraction of the cubic phase dominates. This, however, does not necessarily mean that the mechanical properties of cubic Al(Ti)N are better than those of cubic Ti(Al)N. Since with an increasing thickness of the cubic Al(Ti)N lamellae also lamellar compositions and thereby stabilizing lattice strains are altered, it is not unlikely that the maximal volume fraction of cubic Al(Ti)N leads to the most favourable residual stress state, resulting in the maximum hardness (Zhang, 2015 ▸).

The advantage of the presented new methodological approach is the possibility to screen relatively large thin-film cross sections for novel microstructures as well as phases and to identify promising regions using fast X-ray nanodiffraction. The nanodiffraction scans in Fig. 2 ▸ took less than 1 min each. This opens the possibility for high-throughput combinatorial structure–property refinement in nanomaterials. Although the TEM analysis was very beneficial for the understanding of the particular nanolamellar microstructure (Figs. 3 ▸ and 4 ▸), the actual correlation of the cross-sectional phase evolution (Figs. 2 ▸ *a* and 2 ▸ *b*) and physical properties (Fig. 5 ▸) was achieved by the comparison of the XRD and nanoindentation data. The high-temperature behavior of the novel nanostructure was discussed in our previous report (Todt *et al.*, 2016 ▸).

In the future, it can be expected that the search for novel nanomaterials using cross-sectional synchrotron X-ray nanodiffraction will be performed by analysing thick cross sections of graded thin films, which were deposited by employing the consecutive variation of much more than only one deposition parameter. In this way, (i) combinatorial and high-throughput screening of whole nanomaterial ‘libraries’ will be performed and (ii) deposition conditions resulting in the formation of novel materials will be identified.

## 5. Conclusions   

A set of nanoscale experiments were used to search for the optimum phase composition, microstructure and mechanical properties of nanolamellar TiAlN thin films prepared under specifically selected process conditions. Using an iterative refinement of the structural and functional properties of cross sections of subsequently deposited thin films with progressively narrower structure–function–process windows, it was possible to identify (i) the film composition and microstructure resulting in optimized functional properties of the films and (ii) the corresponding process conditions.

The results demonstrate that, in self-organized TiAlN thin films, Al(Ti)N nanolamellae can spontaneously form in hexagonal and cubic modifications, while Ti(Al)N nanolamellae are always cubic. It has been observed that the film with the maximum hardness can be obtained when the cubic Al(Ti)N lamellae have a maximum thickness of ∼12 nm, while the thickness of Ti(Al)N is ∼1.3 nm. This microstructure has resulted in a hardness of ∼36 GPa.

## Figures and Tables

**Table 1:** Basic process parameters used for synthesis of Ti*_x_*Al_1−*x*_N films

Film label	Gas flow ratio range of AlCl_3_/TiCl_4_	Normalized Ti/Al atomic concentration ratio ranges	Film thickness (µm)
A	2.75–0.6	7:93–50:50	8
B	1.25–2.75	28:72–7:93	9
C	1.9	20:80	3
